# Prevalence, Awareness, Treatment, Control, and Related Factors of Hypertension among Tajik Nomads Living in Pamirs at High Altitude

**DOI:** 10.1155/2020/5406485

**Published:** 2020-07-14

**Authors:** Lin Wang, Nanfang Li, Mulalibieke Heizhati, Suofeiya Abulikemu, Delian Zhang, Qin Luo, Ling Zhou, Jing Hong, Junli Hu, Li Cai, Xin Zhao, Le Sun, Liang Shao

**Affiliations:** ^1^Hypertension Center of People's Hospital of Xinjiang Uygur Autonomous Region, Xinjiang Hypertension Institute;National Health Committee Key Laboratory of Hypertension Clinical Research Urumqi, No. 91 Tianchi Road, Urumqi 830001, Xinjiang, China; ^2^Xinjiang Medical University, Urumqi, Xinjiang Uygur Autonomous Region, China

## Abstract

**Background:**

Hypertension is a global problem, for which high-altitude residents exhibit higher burden. Hypertension in Tajik nomads from Pamirs with an average altitude above 4000 m remains less studied. We aimed to determine the prevalence, awareness, treatment, control, and risk factors associated with hypertension among Tajik population in Pamirs.

**Methods:**

A cross-sectional survey was conducted between August and September 2015 using stratified three-stage random sampling in Taxkorgan county, Pamirs, China. Hypertension is defined as mean systolic and/or diastolic blood pressure (SBP, DBP) ≥140/90 mmHg and/or taking antihypertensive medication within the past two weeks. The prevalence (SBP ≥130 or DBP ≥80 mmHg) was also estimated using the 2017 American College of Cardiology (ACC)/American Heart Association (AHA) High Blood Pressure Guideline. The awareness, treatment, and control of hypertension and associated factors were evaluated.

**Results:**

Totally, 797 subjects aged ≥18 years were enrolled with 46.3% men and 88.8% nomads with the mean age of 42.3 ± 15.2 years. The prevalence of hypertension was 24.2% (140/90 mmHg), and the prevalence was as high as 40.3%, based on the 2017 ACC/AHA guideline. Overall awareness, treatment, and control of hypertension were 52.8%, 40.9%, and 9.3%, respectively. In multivariate logistic regression, BMI ≥24.0 kg/m^2^ (OR: 2.41, 95% CI: 1.44–4.04) was a risk factor for prehypertension, and age ≥60 years (OR: 2.04, 95% CI: 1.15–3.61), BMI ≥24.0 kg/m^2^ (OR: 2.04, 95% CI: 1.15–3.61), and abdominal obesity (OR: 1.87, 95% CI: 1.09–3.22) were risk factors for hypertension. Angiotensin-converting enzyme inhibitors/angiotensin receptor blockers were the most commonly used antihypertensive medication (45.4%) as monotherapy, and 13.6% of treated hypertensive patients used two drugs.

**Conclusions:**

There is a considerable prevalence of hypertension with low awareness, treatment, and control rates among Tajik nomads in Pamirs, where health programs improving the hypertension status are urgently needed, with the excess weight loss as a strategy.

## 1. Introduction

Hypertension is a leading risk factor of cardiovascular disease (CVD), accounting for approximately half of the cardiovascular morbidity and mortality, which has become a serious public health problem in the world, especially in developing countries and regions due to poor management [1–3]. Residents in high-altitude regions with low temperatures and oxygen levels have indigenous diets and lifestyles (e.g., high salt diet, lack of fish, vegetables, and fruits), which can affect the development of hypertension [4, 5]. Several studies in different high altitude areas (i.e., Latin America, USA, Europe, and Asia) show that high-altitude residents exhibit higher risk for hypertension and CVD than low-altitude residents due to the special environment and poor medical conditions [6–8].

People living in the Andean mountains of South America, the Himalayan mountains of Tibet, and the Ethiopian summits of Africa are the three largest populations with the longest history of high altitude residency [9]. The status of hypertension among them has been reported in the last decades [10–12]. However, there are large differences in the magnitude of hypertension among geographic areas and ethnic groups [13, 14]. Little attention has been devoted to determine the magnitude and management of hypertension in the Tajik population living in the Pamirs with an average altitude level of 4,500 meters. Pamirs is located in the southeastern part of Central Asia, the western part of China, and across Tajikistan and China. In addition, the main ethnic resident in Pamirs is Tajik population, who lead nomadic or seminomadic lives usually moving around with seasonal changes in order to maintain the stocks with better resource of fodders, which makes the access of fresh vegetables and fruits further difficult, penetration rate of medical resources low, and health awareness poor. All these factors have relevance with a higher prevalence of hypertension and its poor management [15, 16].

Numerous studies on hypertension have been carried out in China in the last few decades [17–19]. Nonetheless, studies in Pamirs are almost rare. In addition, Tajikistan is located in Northwest part of Pamirs, where the mountains and plateaus account for 90% and about half are above 3,000 meters above sea level, and therefore known as the “mountain country” [20]. The major ethnic group in Tajikistan is also the Tajiks (70%) [20]. However, information about epidemiological investigations of hypertension of populations in Tajikistan is limited, although CVD burden is huge there as well [21].

To explore the magnitude, management, and contributors including the prescription pattern of antihypertensive agents of hypertension among Tajik population in Pamirs, our hypertension center launched an investigation in Taxkorgan county located in the eastern Pamirs with an average altitude of 4000 m and with Tajik population as the main ethnic group, Xinjiang, China. Thereby, the results may provide basis for the design and implementation of appropriate interventions for hypertension in Tajik residents living in Pamirs, extending to Tajikistan with the same ethnicity and approximate conditions in terms of geographical and climatic condition and hypertension-related disease burden.

## 2. Materials and Methods

### 2.1. Study Population

This cross-sectional study was conducted among Tajik residents in Taxkorgan county with an average altitude above 4000 m, China, between August and September 2015. A three-stage (township-village-resident) random sampling method was used to select participants who were aged ≥18 years from the general population. First, seven townships in Taxkorgan county were selected using simple random sampling (SRS). Second, two villages were selected in each townships using SRS. Finally, participants were chosen using SRS according to the population composition. Participants were chosen from the list provided by the local government registers of households. The sample size was based on a prevalence of hypertension of 40% among the population aged ≥18 years from the region with an altitude of at 4000 m [22] and was estimated as 600. Finally, the final sample size was calculated as *N* = 800, considering as high as 25% nonresponse rate.

The eligible criteria for study population were as follows: (a) residents who live permanently in the local; (b) Tajik ethnicity; (c) residents who are willing to cooperate with the investigation (questionnaire interviews and anthropometric measurements). The exclusion criteria were as follows: (a) residents with mental diseases and without clear consciousness; (b) women who are pregnant. The study was approved by the Ethical Review Board of People's Hospital of Xinjiang Uygur Autonomous Region, China.

### 2.2. Training and Data Collection

All study investigators and staff members were trained to be familiar with both the aims of the study and the specific tools and methods used. A standardized questionnaire was administered by trained staff to obtain information on demographic characteristics, health-related behaviors, and hypertension-related information. Anthropometric variables were measured by trained staff after the completion of the questionnaire.

### 2.3. Questionnaire Interviews

Demographic characteristics (sex, age, occupation, education attainment status, personal incomes per family member, marital status, etc.), health-related behaviors (cigarette smoking and alcohol consumption), and hypertension-related information (whether it was previously diagnosed by a doctor? whether it has been treated? whether taking antihypertensive drugs within the previous two weeks?) were investigated by our investigators. Antihypertensive agents were recorded if they were taking them.

### 2.4. Measurements of Blood Pressure

Blood pressure (BP) was measured with the OMRON HBP-1300 Professional Portable Blood Pressure Monitor (OMRON, Kyoto, Japan) three times on the right arm positioned at the heart level after the participant was sitting at rest for five minutes, with 30 seconds between each measurement with an observer present. The average of the three readings was used for the analysis.

### 2.5. Anthropometric Variables

Anthropometric variables were measured using standard equipment and procedures including height, weight, and waist circumference (WC). Height was measured without shoes using a standard right-angle device and a fixed measurement tape (to the nearest 0.1 cm). Body weight without heavy clothing was measured using an OMRON body fat and weight measurement device (V-body HBF-371, OMRON, Kyoto, Japan). WC was measured in the midpoint between the lower rib and upper margin of the iliac crest, measured by a ruler tape with an insertion buckle at one end (to the nearest 0.1 cm) [23]. Body mass index (BMI) was calculated as weight divided by the square of height (kg/m^2^).

### 2.6. Definitions

*Hypertension* is defined as systolic BP (SBP) ≥140 mmHg, and/or diastolic BP (DBP) ≥90 mmHg, and/or use of antihypertensive medicine within two weeks (2010 Chinese Guideline for the Management of Hypertension) [24]. The prevalence of hypertension was also estimated according to the 2017 American College of Cardiology (ACC)/American Heart Association (AHA) High Blood Pressure Guideline [25].

*Prehypertension* is defined as participants who have not been informed of hypertension diagnosis and with SBP 120–139 mmHg and/or DBP 80–89 mmHg [24] and not on antihypertensive drugs treatment.

*Awareness* is defined as whether they have a medical diagnosis of hypertension and *treatment* as whether they are receiving BP-lowering drugs within the past two weeks. *Control* is defined as an average SBP and DBP <140/90 mmHg.

*Education attainment status* is categorized into three levels: primary and lower, junior high and, senior high and higher. *Family income per member* was divided into low incomes ≤¥1500/month (USA$ 217.4/month) and high incomes >¥1500/month (USA$ 217.4/month), based on Chinese individual income tax rates [26]. Marital status was coded as single, married, or widowed/divorced. *Current cigarette consumption* is defined as participants who have smoked at least 20 packets of cigarettes in their lifetime and currently smoke cigarettes and *never* smokers as participants who never smoked or smoked <20 packets of cigarettes in their entire lifetime [17]. *Current alcohol intake* is defined as consuming at least once alcoholic beverage per week in the past month [17]. *Overweight and obesity* are defined as a BMI between 24.0–27.9 kg/m^2^ and of ≥28.0 kg/m^2^, respectively, based on the criteria recommended by the Working Group on Obesity in China (WGOC) [24]. High BMI level (≥24 kg/m^2^) for the current study includes overweight and obesity category. *Abdominal obesity* is defined as WC ≥90 cm in men and WC ≥85 cm in women [27].

Medications were classified as diuretics, beta-blockers, calcium channel blockers (CCBs), angiotensin-converting enzyme inhibitors (ACEIs), angiotensin II receptor blockers (ARBs), and traditional Chinese medicine (TCM). Except for ACEIs and ARBs, each medication was classified into only one category: participants who used ≥2 class drugs as combination therapy.

## 3. Statistical Analysis

Continuous variables were presented as mean and standard deviations (SD) and analyzed using the ANOVA test. Categorical variables were expressed as frequency (*n*) and proportion (%) and analyzed using the chi-squared test. As for prehypertension and hypertension, multiple logistic analysis was used to analyze the associated factors, and adjusted odds ratio (OR) with associated 95% confidence interval (95% CI) was calculated. All statistical tests were two-tailed, and differences were considered statistically significant when the *P* value was <0.05. All statistical analyses were performed using SPSS 20.0 for Windows.

## 4. Results

### 4.1. Population Characteristics

A total of 797 Tajik subjects aged 42.30 ± 15.21 years were enrolled with men accounting for 46.3% (response rate 93.8%). Characteristics of the study population are shown in Table 1. Subjects with stock-raising occupations accounted for 88.8%. The education attainment status of participants was low with over two-thirds (69.1%) having primary or lower education. Regarding the family income per member, the proportion of those with ≤¥1500/month was high (73.1%). Current smoker and current drinkers accounted for 17.4% and 8.6%, respectively. The overall prevalence of overweight and obesity (BMI ≥24.0 kg/m^2^) and abdominal obesity were 54.1% and 45.9%, respectively.

### 4.2. Level of Blood Pressure by Age and Sex

**Figure 1:**
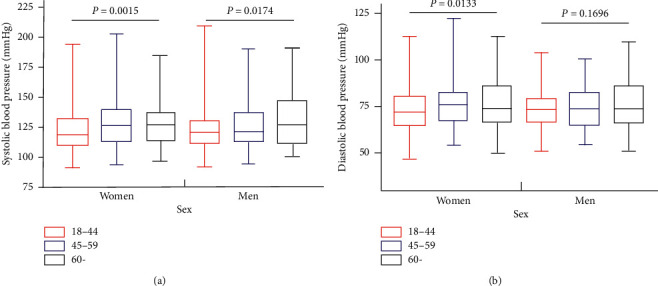
Level of blood pressure by age and sex (a) for systolic blood pressure and (b) for diastolic blood pressure.

Figures 1(a) and 1(b) show the mean BP of all participants by age and sex. Mean SBP and DBP of the participants were (125.7 ± 20.3) mmHg and (74.2 ± 11.5) mmHg, respectively (Supplementary Table 1). SBP and DBP increased significantly with age (*P* < 0.05), while DBP did not show this trend in men.

### 4.3. Prevalence of Prehypertension and Hypertension

The overall prevalence of prehypertension and hypertension was 30.1% (95% CI, 26.8%–33.5%) and 24.2% (95% CI, 21.2%–27.3%), respectively, as shown in Table 2. The prevalence of hypertension, based on the 2017 ACC/AHA guideline, was approximately twice as high as that based on 2010 Chinese guideline (40.3% vs 24.2%). The prevalence of hypertension increased significantly with the advanced age (*P* values for trend = 0.002), while this trend was not observed in the prevalence of prehypertension (*P*=0.894). The prevalence of prehypertension and hypertension showed no significant differences between men and women, as well as between different family income per member groups (*P* > 0.05). The prevalence of hypertension was higher in participants with low education attainment (*P*=0.006), divorced status (*P*=0.003), and BMI ≥24.0 kg/m^2^ (*P* < 0.001).

### 4.4. Awareness, Treatment, and Control of Hypertension

Among those with hypertension, 52.8% (95% CI, 45.7%–59.9%) were aware of their condition, 40.9% (95% CI, 33.9%–47.9%) were taking antihypertensive agents, whereas only 9.3% (95% CI, 5.2%–13.6%) achieved BP control, and control rate among treated hypertensives was 22.7% (95% CI, 15.1%–30.3%) in Table 3.

The awareness and treatment of hypertension significantly increased with age (both *P* values for trend <0.01). Though not reaching statistical significance, the awareness, treatment, and control of hypertension were lower among population with low family incomes than their counterparts (51.8% vs. 56.5%, 36.9% vs. 44.3%, 6.6% vs.11.1%, all *P* values >0.05), and the awareness of hypertension was lower in population with single marital status than married and divorce status (25.0% vs. 51.9%, 68.9%, *P* value = 0.033). There were no significant differences in the awareness, treatment, and control of hypertension between genders (all *P* values >0.05).

### 4.5. Multivariable Risk Assessment

Tables 4 and 5 show the results of multiple logistic regression analysis of associations of potential risk factors with prehypertension and hypertension among participants, respectively. Higher BMI (OR: 2.41, 95% CI: 1.44–4.04) could increase prevalence of prehypertension. Age ≥60 years (OR: 2.04, 95% CI: 1.15–3.61), BMI ≥24.0 kg/m^2^ (OR: 2.04, 95% CI: 1.15–3.61), and abdominal obesity (OR: 1.87, 95% CI: 1.09–3.22) were significantly associated with the presence of hypertension.

### 4.6. Pattern of Antihypertensive Agent Usage

A total of 74 hypertensive individuals were reported taking antihypertensive drugs at the time of the survey, whereas 59.5% (*n* = 44) of the subjects provided the detailed information on drug use (Table 6), of whom, 45.4% were taking ACEI/ARBs, 31.8% taking CCBs, 11.3% beta blockers, 11.3% diuretics, and 13.6% TCM. While further analyzing the combination therapy, it was observed that 13.6% of hypertensive individuals were prescribed for two classes drugs and no patients prescribed for three or more drugs.

## 5. Discussion

This survey is the first in Tajik population living in Pamirs with an altitude of at least 4000 m China to report the hypertension status in relatively representative population aged ≥18 years including nearly 90% stock raisers. The prevalence of hypertension is 24.2%, with poor awareness, treatment, and control rates. A minority of hypertensive individuals is treated, and less than 1 in 10 hypertensives have their BP controlled. In addition, control rate of hypertension among treated individuals is less than one quarter. Less than one-seventh receives two or more drugs at the time of survey.

Some studies on hypertension in high-altitude residents have been carried out in the last few decades [10–12]. The prevalence of hypertension at different high altitude locations ranges from 8.6% to 55.9% [28, 29]. A meta-analysis synthesizing high altitude prevalence studies suggests an altitude gradient in the prevalence of hypertension. That is, the prevalence increases with increasing altitude [22]. In addition, the prevalence of hypertension is 44.6% and 41.4% in Tibetan and non-Tibetan populations living at altitude of ≥4000 m, respectively [22]. Nonetheless, the prevalence is 24.2% in Tajik residents in the Pamirs plateau at an altitude of ≥4,000 m. The current study is unable to explain the inconsistency in the prevalence, whereas it is possibly related to following factors. First, the Tajik people may have a better adaptation to the high-altitude hypoxic environment, in terms of genetic susceptibility of the population and adaptability to high-altitude hypoxia [9, 30, 31], which may imply that ethnic and regional differences may cause diversity in the prevalence of hypertension. However, further studies on interaction mechanism of hypertension between the genetic and environmental and strength of the relationships are warranted on this aspect. Second, the minimum age to participate in the present study was 18 years, whereas other studies usually included participants from the age of 40 years or over [29, 32]. Nonetheless, hypertension increases in prevalence with the age.

The current study shows different results compared with studies including the same age groups at other high altitudes. For instance, a recent survey of hypertension and diabetes mellitus conducted in Uttarakhand, India (altitude of 2084 m), found that 54.5% of participants aged ≥60 years have hypertension [10]. This contrasts with our study's 34.2% prevalence of hypertension. Divergent results may partly be explained by characteristics of researches, including differences in region, ethnicity, lifestyle, and sampling methodology. For instance, participants in our survey are randomly selected, with selection bias less than that conducted in door-to-door-based study in Uttarakhand. When the door-to-door method is used to select the samples, persons who stay at home due to chronic diseases are more likely to be selected, which may increase the prevalence of hypertension in the entire study. Moreover, Uttarakhand is mainly occupied Brahmin and Kshatriya populations [33], mainly from east Eurasia [34] and may have different genetic background, compared with Tajiks. Therefore, different ethnicity can also have a role on the prevalence of hypertension [35, 36].

The current study contains some information compared with the national hypertension survey. The prevalence of hypertension in Tajik nomads living in Pamirs with altitude of ≥4000 meter is similar to the national prevalence of China for the population aged ≥18 years (23.2%) [17]. Additionally, the average SBP and DBP levels of the sampled population are consistent with the national average. This may be because long-term residents of high altitude do not seem to differ from others in resting BP in non-Tibetans [22]. Furthermore, in the present study, only 17.4% and 8.6% of the participants self-reported to be current smokers and drinkers, respectively, which is less than the national prevalence of 20.6% and 15.8% for the Chinese population aged ≥18 years [17]. As a result, the influence of the altitude may have been obscured by lifestyle-related risk changes. Nevertheless, in the present study, the control of hypertension was very poor, compared with the national control rate (9.3% vs. 15.3%). Background possible reasons may include the following. First, medications are not readily available in stock-raising regions [37], and larger proportions of stock raisers could not afford the drugs [38, 39]. Furthermore, hypertensives stop taking agents when BP is controlled, resulting in uncontrolled BP when checked later [40]. Second, agent selection at high-altitude regions has impacts on BP control as well. For instance, BP-lowering efficacy of ARBs is poor reportedly at higher altitude [41]. Nonetheless, ARB/ACEI accounts for 45.4% in current treated subjects. Moreover, prescription patterns of village care-providers are different from those of tertiary hospital physicians [40]. In fact, only 13.6% of hypertensives in the survey received combination therapy, possibly standing for one of the reasons for poorly managed hypertension here.

Therefore, our findings highlight the need for developing a region-targeted hypertension education program to coordinate the efforts of detection, prevention, and treatment of hypertension in plateau regions. In addition, considering the limited availability of antihypertensive drugs and the limited affordability of locals, especially of nomads, exploring and promoting simplified, easy-to-master and cost-effective antihypertensive algorithms might be the good pathway for hypertension control.

BMI and body weight are the best predictors of higher BP in high altitude residents [22]. This is consistent with current results that the correlation with prehypertension and hypertension is higher among overweight and obese subjects than others. The mechanism by which obesity is linked with hypertension is not properly understood. However, an increase in insulin resistance, sympathetic activity, hyperleptinemia, renal abnormalities, and sodium retention is thought to be the likely underlying pathway [42]. Therefore, control of BMI is a critical public health priority in prevention of hypertension locally as well.

The Tajik residents mainly live in the Pamirs and are distributed in Taxkorgan county, Xinjiang, China, and Tajikistan, who still share life style and geographical and climatic environment and burden of CVD including hypertension. Therefore, results of the current study could extend to Tajik population from Tajikistan population living in a similar altitude and nearby areas in terms of prevention of hypertension.

## 6. Strengths and limitations

Current analysis is strengthened by relatively representative study subjects from the Tajik ethnic group, which makes the report one of the valuable information for public health sectors and for clinical setting. However, this study contains some limitations. First of all, the cross-sectional nature of the study does not allow to get a cause-and-effect relationship between hypertension and related factors. However, it is also the common way of finding problems and providing clues for prevention. Second, current analysis failed to focus on the data of salt intake, blood lipid, and glucose, which might have brought some bias on analysis of associated factors of prehypertension and hypertension. However, their relationship with hypertension is well-established and still the focus when providing individualized treatment or in population level programs. Third, this study had a relatively small sample size, which may have diminished the statistical power for subgroup analysis. Fourth, we tried to provide information on the drug use pattern here, whereas approximately 40% of hypertensive subjects lacked of data on this aspect, which may have had some information bias. This observation needs further confirmation in studies with well-powered sample size.

## 7. Conclusions

Prevalence of hypertension is considerable among Tajik nomads aged ≥18 years living in Pamirs with an altitude of ≥4000 m, China with poor awareness, treatment and control rates. Excess weight loss is a vital strategy for prevention of hypertension. In addition, improving the access of primary care, strengthening the ability construction of medical teams and exploring optimized anti-hypertensive algorithms are crucial to improve the hypertension status in the stock-raising region of Pamirs. Current results could also extend to Tajik population from Tajikistan population living similar altitude and nearby areas.

## Figures and Tables

**Table 1:** Baseline characteristics of the study population by sex.

Variables	Men (*n* = 369)	Women (*n* = 428)	Total (*n* = 797)	*P*
Age (years)	42.19 ± 15.21	42.40 ± 15.23	42.30 ± 15.21	0.849
18–44	224 (60.7)	254 (59.3)	478 (60.0)	
45–59	93 (25.2)	115 (26.9)	208 (26.1)	0.867
≥60	52 (14.1)	59 (13.8)	111 (13.9)	
Stock raisers (*n*, %)	325 (88.1)	383 (89.5)	708 (88.8)	0.601
Education attainment status (*n*, %)				
Primary and lower	215 (58.4)	336 (78.3)	551 (69.1)	
Junior high	78 (21.2)	66 (15.4)	144 (18.1)	<0.001
Senior high and higher	75 (20.4)	27 (6.3)	102 (12.8)	
Family income per member (*n*, %)				0.001
Low	247 (66.9)	336 (78.5)	583 (73.1)	<0.001
High	122 (33.1)	92 (21.5)	214 (26.9)	
Marital status (*n*, %)				
Single	48 (13.0)	28 (6.5)	76 (9.5)	<0.001
Married	298 (80.8)	348 (81.3)	646 (81.1)	
Widowed/divorced	23 (6.2)	52 (12.2)	75 (9.4)	
Current cigarette consumption (*n*, %)	137 (37.1)	2 (0.5)	139 (17.4)	<0.001
Current alcohol intake (*n*, %)	69 (18.7)	0 (0)	69 (8.6)	—
Body mass index				
<23.9 kg/m^2^	173 (46.9)	193 (45.1)	366 (45.9)	0.619
≥24.0 kg/m^2^	196 (53.1)	235 (54.9)	431 (54.1)	
Waist circumference				
Abdominal obesity (*n*,%)	135 (36.6)	200 (46.7)	335 (42.0)	0.003

**Table 2:** Prevalence of prehypertension and hypertension in the study population.

Stratification	PHT (*n* = 240) (%) (95% CI)	HT^1^ (*n* = 193) (%) (95% CI)	HT^2^ (*n* = 321) (%) (95% CI)
Sex			
Men	30.1 (25.6–34.9)	23.6 (19.5–28.2)	39.1 (34.5–43.8)
Women	30.1 (26.2–34.7)	24.8 (20.8–29.0)	40.8 (35.1–46.2)
*P* value	1.000	0.740	0.613
Age			
18–44	29.9 (25.6–34.3)	20.1 (16.7–23.9)	33.3 (23.9–33.9)
45–59	31.2 (25.3–37.9)	28.4 (22.6–38.9)	49.0 (41.2–51.7)
≥60	28.8 (21.1–37.9)	34.2 (25.9–43.6)	54.1 (50.1–61.6)
*P* value	0.894	0.002	<0.001
Education attainment status			
Primary and lower	28.7 (24.6–32.7)	26.5 (22.9–30.3)	42.1 (37.1–47.0)
Junior high	35.4 (28.0–43.6)	22.9 (16.7–30.5)	39.9 (34.3–45.2)
Senior high and higher	30.4 (22.2–40.0)	13.7 (8.2–21.9)	28.8 (23.1–34.3)
*P* value	0.297	0.006	0.017
Family income per member			
Low (reference)	30.5 (25.6–35.3)	23.8 (18.0–29.5)	41.1 (35.4–46.2)
High	28.9 (24.8–33.1)	25.2 (18.2–32.3)	39.2 (34.7–45.9)
*P* value	0.764	0.602	0.926
Marital status			
Single (reference)	36.8 (26.7–48.3)	15.8 (9.1–25.8)	31.2 (25.6–36.8)
Married	28.9 (25.1–32.8)	23.5 (20.3–26.9)	38.5 (33.5–43.7)
Divorced	33.3 (23.6–44.8)	38.7 (28.3–50.2)	63.2 (56.2–70.1)
*P* value	0.291	0.003	<0.001
Body mass index			
<23.9 kg/m^2^ (reference)	19.4 (15.3–23.5)	13.7 (10.1–17.2)	22.1 (17.8–26.4)
≥24.0 kg/m^2^	39.2 (34.6–43.8)	33.2 (28.7–37.6)	55.7 (50.9–60.4)
*P* value	<0.001	<0.001	<0.001
Overall	30.1 (26.8–33.5)	24.2 (21.2–27.3)	40.3 (36.1–44.1)

PHT, prehypertension; HT, hypertension; CI, confidence intervals.^1^ Based on the criteria of 2010 Chinese High Blood Pressure Guideline.^2^ Based on the criteria of 2017 ACC/AHA High Blood Pressure Guideline.

**Table 3:** Awareness, treatment, and control of hypertension in the study population.

	Awareness (*n* = 102) (%) (95% CI)	Treatment (*n* = 79) (%) (95% CI)	Control (*n* = 18) (%) (95% CI)	Control in the treated (*n* = 18) (%) (95% CI)
Sex				
Men	50.6 (39.8–61.2)	41.3 (30.8–51.9)	9.2 (3.0–15.4)	22.3 (13.7–31.0)
Women	54.7 (45.1–64.3)	40.5 (31.1–50.1)	9.4 (3.8–15.1)	23.2 (9.5–37.2)
*P* value	0.664	0.909	0.995	0.913
Age				
18–44	35.4 (25.7–45.2)	29.2 (19.9–38.4)	6.2 (1.3–11.1)	21.2 (9.5–32.8)
45–59	64.4 (51.8–76.9)	54.2 (41.1–67.3)	10.2 (2.2–18.1)	18.8 (5.2–32.4)
≥60	78.9 (65.4–92.5)	50.0 (33.3–66.7)	15.8 (3.6–27.9)	31.6 (22.6–40.5)
*P* value	＜0.001	0.004	0.223	0.560
Education attainment status				
Primary and lower	56.4 (48.7–64.1)	41.7 (33.9–49.8)	10.7 (5.8–15.6)	25.6 (14.3–36.8)
Junior high	39.4 (21.7–56.1)	33.3 (16.2–50.6)	5.9 (2.5–9.3)	17.7 (8.4–27.1)
Senior high and higher	50.0 (20.0–79.9)	50.0 (38.2–61.7)	6.2 (1.5–10.9)	12.4 (2.9–21.8)
*P* value	0.214	0.520	0.314	0.271
Family income per member				
Low	51.8 (41.9–60.8)	36.9 (27.8–46.1)	6.6 (1.3–11.9)	17.9 (7.6–28.1)
High	56.5 (40.4–70.6)	44.3 (29.3–59.5)	11.1 (1.5–20.5)	25.1(13.2–37.1)
*P* value	0.724	0.469	0.778	0.879
Marital status				
Single	25.0 (3.7–46.3)	21.0 (3.4–38.7)	—	—
Married	51.9 (43.6–59.3)	42.1 (34.1–50.0)	9.2 (2.6–15.5)	21.8 (11.9–31.6)
Divorced	68.9 (51.0–86.8)	41.3 (22.3–60.4)	10.0 (2.9–17.1)	24.2 (4.2–44.1)
*P* value	0.033	0.509	0.516	0.627
Body mass index				
<23.9 kg/m^2^	48.0 (33.6–62.3)	32.0 (18.6–45.4)	12.0 (2.6–21.3)	37.5 (24.5–50.6)
≥24.0 kg/m^2^	54.5 (46.3–62.8)	44.1 (35.8–52.3)	8.4 (3.7–12.9)	19.0 (4.4–33.6)
*P* value	0.511	0.181	0.572	0.179
Overall	52.8 (45.7–59.9)	40.9 (33.9–47.9)	9.3 (5.2–13.6)	22.7 (15.1–30.3)

Control is defined as BP <140/90 mmHg; CI, confidence intervals.

**Table 4:** Factors associated with prehypertension from study population by multiple logistic regression.

Stratification	*N* (%)	PHT OR (95% CI)	*P* value
Sex			
Men	111/369 (30.1)	1 (reference)	0.458
Women	129/428 (30.1)	1.19 (0.76–1.87)	
Age			
18–44	143/478 (29.9)	1 (reference)	
45–59	65/208 (31.2)	0.92 (0.60–1.40)	0.687
≥60	32/111 (28.8)	0.65 (0.35–1.19)	0.160
Education attainment status			
Primary and lower	158/551 (28.7)	1 (reference)	
Junior high	51/144 (35.4)	1.49 (0.91–2.45)	0.113
Senior high and higher	31/102 (30.4)	1.19 (0.68–2.09)	0.532
Family income per member			
Low	178/583 (30.5)	1 (reference)	0.540
High	62/214 (28.9)	0.88 (0.58–1.34)	
Marital status			
Single	28/76 (36.8)	1 (reference)	
Married	187/646 (28.9)	1.05 (0.52–2.10)	0.897
Divorced	25/75 (33.3)	1.14 (0.43–3.01)	0.798
Body mass index			
BMI: <23.9 kg/m^2^	71/366 (19.4)	1 (reference)	＜0.001
BMI: ≥24.0 kg/m^2^	169/431 (39.2)	2.41 (1.44–4.04)	
Waistline circumference			
Normal	111/462 (24.0)	1 (reference)	0.319
Abdominal obesity	129/335 (38.5)	1.29 (0.78–2.12)	
Current cigarette consumption			
No	198/658 (30.1)	1 (reference)	0.420
Yes	42/139 (30.2)	1.25 (0.73–2.13)	
Current alcohol intake			
No	215/728 (29.5)	1 (reference)	0.156
Yes	25/69 (36.2)	1.56 (0.84–2.90)	

Adjusted factors include all above variables. PHT, prehypertension; OR: odd ratio; CI: confidence interval.

**Table 5:** Factors associated with hypertension from study population by multiple logistic regression.

Stratification	*N* (%)	HT OR (95% CI)	*P* value
Sex			
Men	87/369 (23.6)	1 (reference)	0.281
Women	106/428 (24.8)	0.77 (0.48–1.24)	
Age			
18–44	96/478 (20.1)	1 (reference)	
45–59	59/208 (28.4)	1.53 (0.97–2.40)	0.067
≥60	38/111 (34.2)	2.04 (1.15–3.61)	0.015
Education attainment status			
Primary and lower	146/551 (26.5)	1 (reference)	
Junior high	33/144 (22.9)	0.87 (0.50–1.52)	0.633
Senior high and higher	14/102 (13.7)	0.43 (0.22–0.87)	0.019
Family income per member			
Low	139/583 (23.8)	1 (reference)	0.399
High	54/214 (25.2)	1.21 (0.78–1.88)	
Marital status			
Single	12/76 (15.8)	1 (reference)	
Married	152/646 (23.5)	0.91 (0.40–2.12)	0.834
Divorced	29/75 (38.7)	1.72 (0.60–4.96)	0.313
Body mass index			
BMI: <23.9 kg/m^2^	50/366 (13.7)	1 (reference)	0.014
BMI: ≥24.0 kg/m^2^	143/431 (33.2)	2.04 (1.15–3.61)	
Waistline circumference			
Normal	68/462 (14.7)	1 (reference)	0.023
Abdominal obesity	125/335 (37.3)	1.87 (1.09–3.22)	
Current cigarette consumption			
No	157/658 (23.8)	1 (reference)	0.432
Yes	36/139 (25.9)	1.27 (0.70–2.31)	
Current alcohol intake			
No	174/728 (23.9)	1 (reference)	0.284
Yes	19/69 (27.5)	1.52 (0.71–3.24)	

Adjusted factors include all above variables; HT, hypertension; OR: odds ratio; CI: confidence interval; the diagnosis of hypertension is based on the criteria of 2010 Chinese High Blood Pressure Guideline.

**Table 6:** Use of antihypertensive medications in the treated hypertensives.

Type of antihypertensive drug	*N*	(%)
ACEI/ARBs	20	45.4
CCBs	14	31.8
Beta blockers	5	11.3
Diuretics	5	11.3
TCM	6	13.6
Two classes	6	13.6

ACEI, angiotensin-converting enzyme inhibitors. ARB, angiotensin receptor blockers. CCB, calcium channel blockers. TCM, traditional Chinese medicine.

## Data Availability

Materials included in the manuscript, excluding the relevant raw data, will be made freely available to any researchers who wish to use them for noncommercial purposes, while preserving any necessary confidentiality and anonymity.

## Supplementary Materials

Supplementary Materials Supplementary Table 1: mean systolic and diastolic blood pressure by age and sex.
