# Supplementary material for: Prevalence, Awareness, Treatment, Control, and Related Factors of Hypertension among Tajik Nomads Living in Pamirs at High Altitude
Source: Int J Hypertens. 2020 Jul 14;2020:5406485. doi: 10.1155/2020/5406485 (PMC7376436; doi:10.1155/2020/5406485)
Supplement: Supplementary Materials — Supplementary Table 1: mean systolic and diastolic blood pressure by age and sex. [file 5406485.f1.pdf]

Supplementary -Table 1. Mean systolic and diastolic blood pressure by age and sex

Age group	SBP		P value	DBP		P value	Total	
	Male	Female		Male	Female		SBP	DBP
18-44	123.3±18.5	122.8 ±18.3	0.786	73.2±10.8	72.7±11.1	0.626	123.0±18.4	72.9±10.9
45-59	127.3±20.9	130.4±23.4	0.322	74.7±10.8	76.2±12.3	0.316	129.1±22.4	75.5±11.7
≥60	131.6±24.6	128.9±18.5	0.512	76.3±13.7	76.0±11.9	0.895	130.2±21.5	76.2±12.7
P value	0.017	0.002	-	0.013	0.169	-	< 0.001	0.004
Total	125.5±20.1	125.7±20.1	0.873	74.2±11.3	74.3±11.8	0.931	125.6±20.2	74.1±11.5

SBP, systolic blood pressure; DBP, diastolic blood pressure
